# Nanoscale Chemical and Electrical Stabilities of Graphene-covered Silver Nanowire Networks for Transparent Conducting Electrodes

**DOI:** 10.1038/srep33074

**Published:** 2016-09-13

**Authors:** Seong Heon Kim, Woon Ih Choi, Kwang Hee Kim, Dae Jin Yang, Sung Heo, Dong-Jin Yun

**Affiliations:** 1 Samsung Advanced Institute of Technology, Gyeonggi-do 443-803, Republic of Korea

## Abstract

The hybrid structure of Ag nanowires (AgNWs) covered with graphene (Gr) shows synergetic effects on the performance of transparent conducting electrodes (TCEs). However, these effects have been mainly observed via large-scale characterization, and precise analysis at the nanoscale level remains inadequate. Here, we present the nanoscale verification and visualization of the improved chemical and electrical stabilities of Gr-covered AgNW networks using conductive atomic force microscopy (C-AFM), Auger electron spectroscopy (AES), and X-ray photoelectron spectroscopy (XPS) combined with the gas cluster ion beam (GCIB) sputtering technique. Specifically by transferring island Gr on top of the AgNW network, we were able to create samples in which both covered and uncovered AgNWs are simultaneously accessible to various surface-characterization techniques. Furthermore, our *ab initio* molecular dynamics (AIMD) simulation elucidated the specific mechanistic pathway and a strong propensity for AgNW sulfidation, even in the presence of ambient oxidant gases.

For decades, the transparent conducting electrode (TCE) materials used in next-generation electronic devices, including organic thin-film transistors (OTFTs), organic photovoltaics (OPV), dye-sensitized solar cells (DSSCs), and organic light-emitting diodes (OLEDs), have been widely studied to replace the existing transparent conducting oxide (TCO) materials indium-tin-oxide (ITO), metal-doped Zn oxide, and F-doped Sn oxide (FTO)^1^‒^3^. Various types of materials, such as conducting polymer composites, graphene (Gr), and carbon nanotubes (CNTs), have been examined as candidate TCE materials^4^,^5^. However, notwithstanding their outstanding merits of flexibility and solution processability at normal pressure, the sheet resistances (R_S_) and transparencies (T%) of these materials remain insufficient compared with those of existing TCO films (ITO: R_S_ > 50 Ω/□ at T%: 85%). Recently, the network films of one-dimensional metal nanostructures have drawn increasing attention as alternative flexible transparent electrodes because of their excellent electrical conductivity and flexibility^6^‒^11^. In particular, some research groups have already reported the preparation processes for silver nanowire (AgNW) films with similar or higher electrical conductivity than ITO films under the same film-transparency conditions^7^,^8^.

Nevertheless, AgNWs have intrinsic shortcomings that must be overcome before they can be applied as TCEs. One such shortcoming is AgNWs’ susceptibility to corrosion. In addition to oxidation, sulfidation is also a dominant corrosion process affecting Ag-based materials exposed to the atmosphere^12^‒^17^. According to the literature, the main product resulting from the corrosion of AgNWs exposed to the atmosphere is Ag sulfide, as confirmed by the observation of a Ag sulfide layer on AgNWs after storage in air for several weeks by transmission electron microscopy (TEM) and energy-dispersive X-ray spectrometry (EDS)^17^. Indeed, the atmospheric sulfidation process of Ag-based materials is not a self-explanatory phenomenon because S-containing species are rare in air. This report demonstrates that H_2_S and OCS gases are the principal corrodents of AgNW among the reduced-S gases present in the atmosphere, such as H_2_S, OCS, SO_2_, and CS_2_, because they exhibit sulfidation rates that are approximately one order of magnitude higher than those of SO_2_ and CS_2_^13^,^17^. Furthermore, it is emphasized that despite the low concentrations of these gases in air, they are sufficiently abundant to initiate the sulfidation process^16^,^17^. Among these gases, H_2_S is responsible for the main sulfidation process, as shown in Equation (1)^17^.


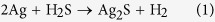


OCS, which is the most abundant S-containing gas in the atmosphere, reacts with water to produce H_2_S, and the sulfidation process of H_2_S is enhanced by the presence of other gases, such as O_2_ and NO_2_, as shown in Equations (2–4)^17^.


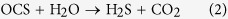



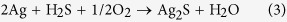



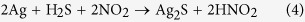


The Gr-AgNW hybrid structure has been proposed to overcome the limitation of AgNW networks because the Gr layer is an atomically thin two-dimensional sheet with outstanding conductivity, transparency, and flexibility^18^‒^29^. Indeed, Lee *et al.* reported that sandwich-structured Gr/AgNW/Gr films show enhanced thermal oxidation stability and improved electrical properties compared with unmixed AgNW films based on long-term stability tests at a high temperature (70 °C) and high relative humidity (70% RH)^26^. Additionally, many studies have demonstrated that Gr can act as an efficient diffusion barrier against various gases and as a protective layer against the oxidation of metal species, such as Ag and Cu^29^‒^36^. Furthermore, Gr has been proposed as a versatile protective layer against bacterial contamination in biomedical applications and as a plasmonic enhancer in plasmonic devices^37^‒^39^. Recently, single-layer Gr was also reported to shield AgNWs from intense ultraviolet (UV) laser-induced damage^29^. In this study, we examined the performance of Gr as a gas barrier to protect AgNWs from corrosion, including sulfidation, and verified the chemical and electrical stabilities of Gr-covered AgNW network on the nanoscale. In particular, we introduced a hybrid structure of island Gr (iGr) and AgNWs to clearly compare the differences between Gr-covered and bare AgNWs.

## Results and Discussion

**Figure 1:**
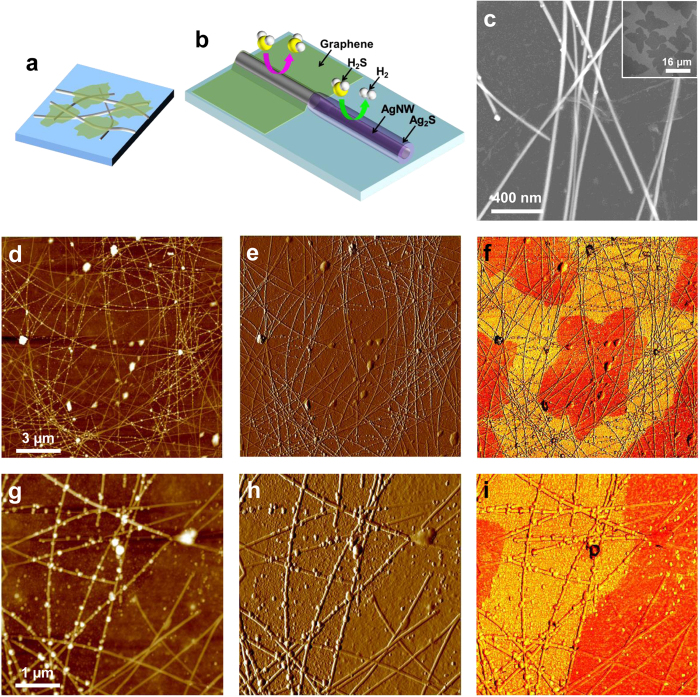
(**a**) Structure of the iGr/AgNW sample. (**b**) Illustration depicting a Gr sheet acting as a protective layer against the sulfidation of AgNWs. (**c**) SEM image of iGr/AgNW after 1 week. The inset is a SEM image of iGrs. (**d**,**g**) Topographical, (**e**,**h**) differentiation, and (**f**,**i**) phase images of iGr/AgNW after 3 weeks.

Figure 1a depicts the structure of the iGr-covered AgNWs (iGr/AgNW) sample that was the focus of this study, and the scanning electron microscopy (SEM) and atomic force microscopy (AFM) images of a pristine iGr layer are shown in the inset of Fig. 1c and Supplementary Fig. 1b, respectively. The iGr layer, which consists of isolated star-shaped Gr islands, can be formed by stopping the Gr layer’s growth before it covers the whole substrate area during a typical growth process for large-area Gr. The resulting iGr layer can be transferred routinely onto various substrates, including polyimide (PI) or polyethylene terephthalate (PET) films used as base substrates in this study. Figure 1c shows a SEM image of iGr/AgNW in which an iGr layer with a folded edge is faintly visible. The prepared iGr/AgNW samples were stored for several weeks in ambient conditions in the laboratory (temperature and RH maintained at approximately 25 °C and 30% RH, respectively). The samples’ morphological, chemical, and electrical changes were traced using various tools, such as AFM, conductive AFM (C-AFM), X-ray photoelectron spectroscopy (XPS), and Auger electron spectroscopy (AES) combined with an Ar gas cluster ion beam (GCIB) sputtering technique. Figure 1d‒i show the AFM imaging results at two different scales for the iGr/AgNW sample kept in ambient atmosphere for 3 weeks. AFM phase imaging is an efficient method to clearly define the iGr-covered area on the iGr/AgNW sample surface, as shown in Fig. 1f,i. The dark orange-colored area indicates the iGr-covered AgNWs, and the bright orange corresponds to bare AgNWs in the phase images (Fig. 1f,i). In the AFM topographic images (Fig. 1d,g), nanoparticle-like structures, which are typically observed on sulfidized AgNWs^17^, were generally newly grown on the bare AgNWs, whereas the iGr-AgNWs were protected, with nanoparticles occurring only rarely. Therefore, it can be concluded that the bare AgNWs were considerably sulfidized and that the iGr-covered ones were protected by the iGr layer, as depicted in Fig. 1b, because the growth of Ag_2_S nanoparticles is characteristic on sulfidized AgNWs exposed to the atmosphere. Furthermore, the bare AgNWs, which were not protected by the iGr layer, were disconnected and eventually ruined by severe sulfidation after being stored in the atmosphere for longer durations, as shown in Supplementary Fig. 2. To verify the exact role of the iGr layer, we performed a similar AFM experiment using AgNW/iGr samples in which the iGr layer was placed under the AgNWs and all AgNWs were exposed to the atmosphere. As shown in Supplementary Fig. 3, the growth of sulfide nanoparticles was not affected by the existence of the underlying iGr layer, and sulfidation occurred homogeneously and randomly on the whole sample areas of the AgNW/iGr samples. Therefore, the iGr layer acts as a barrier layer against the sulfidation of AgNWs and is not directly involved in any chemical reaction that hinders sulfidation.

**Figure 2:**
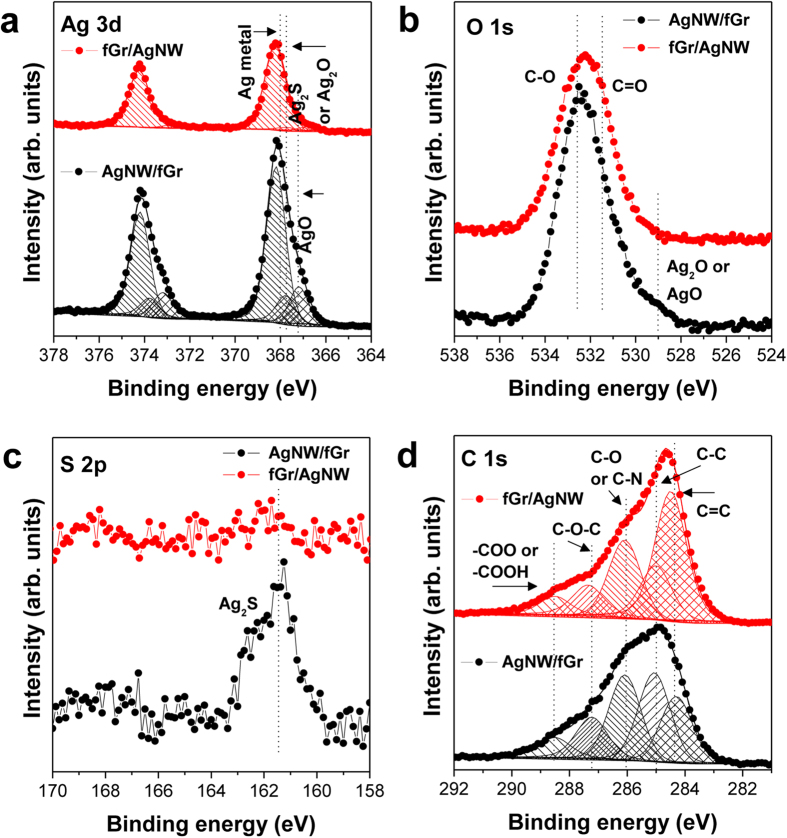
Comparative XPS spectra ((**a**) Ag 3d, (**b**) O 1 s, (**c**) S 2 p, and (**d**) C 1 s core-level structures) of fGr/AgNW and AgNW/fGr samples after identical storage in air for 5 weeks.

To examine whether the Gr layer acts as chemically protective layer against AgNW sulfidation, we additionally prepared other types of Gr-AgNW hybrid structures with large-area Gr sheets (fGr) which can fully cover sample surfaces. According to the position of fGr layer relative to the AgNWs, those are classified into fGr/AgNW (AgNW network fully covered with a large-area Gr) and AgNW/fGr (AgNW network spread over a large-area Gr). These hybrid structures were then analyzed by XPS after storage in the atmosphere for 3 weeks. Figure 2a‒d, which presents the comparative XPS spectra of the fGr/AgNW and AgNW/fGr samples, indicate an enormous difference in the chemical states of AgNWs depending on the position of the Gr layer relative to the AgNWs. As shown in Fig. 2a, the AgNWs in the fGr/AgNW sample completely retained their inherent metallic states (Ag 3d_5/2_: 368.2 eV) with loss of the satellite peak of the AgNWs, whereas the AgNWs in the AgNW/fGr sample also exhibit both the AgO (Ag 3d_5/2_: 367.0 eV) oxidation state and the Ag_2_S sulfidation state (Ag 3d_5/2_: 367.8 eV)^40^,^41^. Accordingly, unlike fGr/AgNW, AgNW/fGr exhibits AgO oxidation (O 1s: 529 eV) and Ag_2_S sulfidation (S 2p: 161.3 eV) states in its O 1s and S 2p spectra, respectively. Such significant differences in the chemical states directly support the efficiency of the Gr layer as a physical barrier layer against the sulfidation and oxidation of AgNWs. Furthermore, the C 1s spectrum of fGr/AgNW in Fig. 2d has a relatively high area ratio of the C = C peak (284.0 eV) to the C-C peak (248.8 eV) compared with that of AgNW/fGr. This difference stems from the position of the Gr layer relative to the AgNWs, and thus, this finding indicates that the Gr layer of the C = C sp_2_ hybridized bonds completely covered the AgNWs on the surface of the fGr/AgNW sample without variation in its chemical structure^5^.

**Figure 3:**
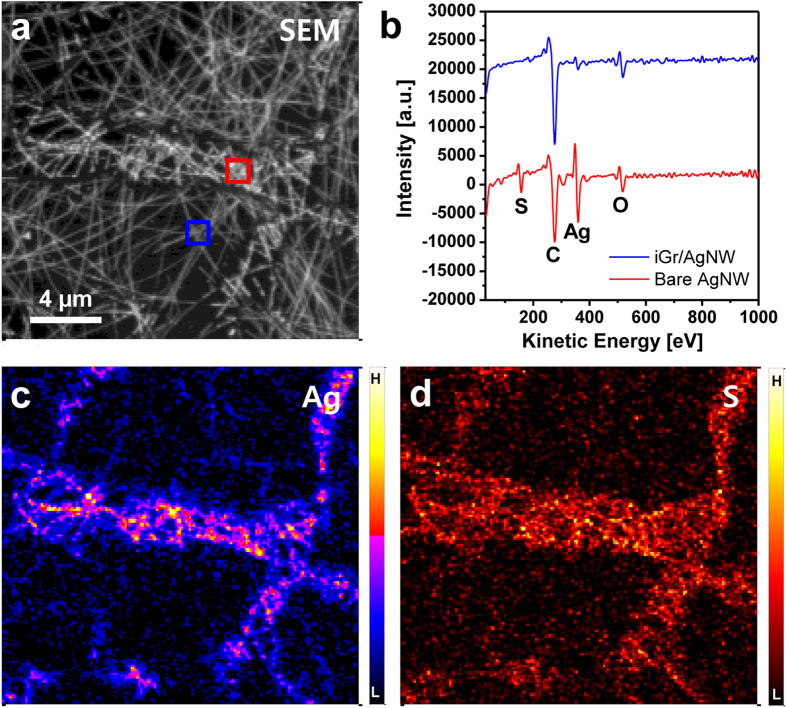
AES measurements of iGr/AgNW after 5 weeks: (**a**) SEM image, (**b**) AES spectra, and (**c**) Ag and (**d**) S mapping images.

Figure 3 shows the AES results obtained for the iGr/AgNW sample after storage in the atmosphere for 5 weeks. The iGr-covered area on the iGr/AgNW sample can be distinguished in the SEM image (Fig. 3a) according to the differences in the morphology and contrast between iGr-covered and bare AgNWs regions. The AES spectra obtained from the iGr-covered and bare AgNW regions (the red and blue rectangular areas, respectively, shown in Fig. 3a) are presented in Fig. 3b. Ag and S exhibit intense signals in the bare AgNW region, and C and O are consistently detected, confirming the AFM result that in the absence of the iGr layer, AgNWs undergo considerable sulfidation. In contrast, S is barely detected and the Ag peak is reduced in the iGr-covered region. This result is in good agreement with the elemental mapping images of Ag and S in Fig. 3c,d. The reduced detection of Ag in the iGr-covered AgNW region results from interference from the iGr layer because AES measurements are extremely surface sensitive and acquire chemical information from a typical probing depth of ~3 nm during AES acquisition without sputtering.

**Figure 4:**
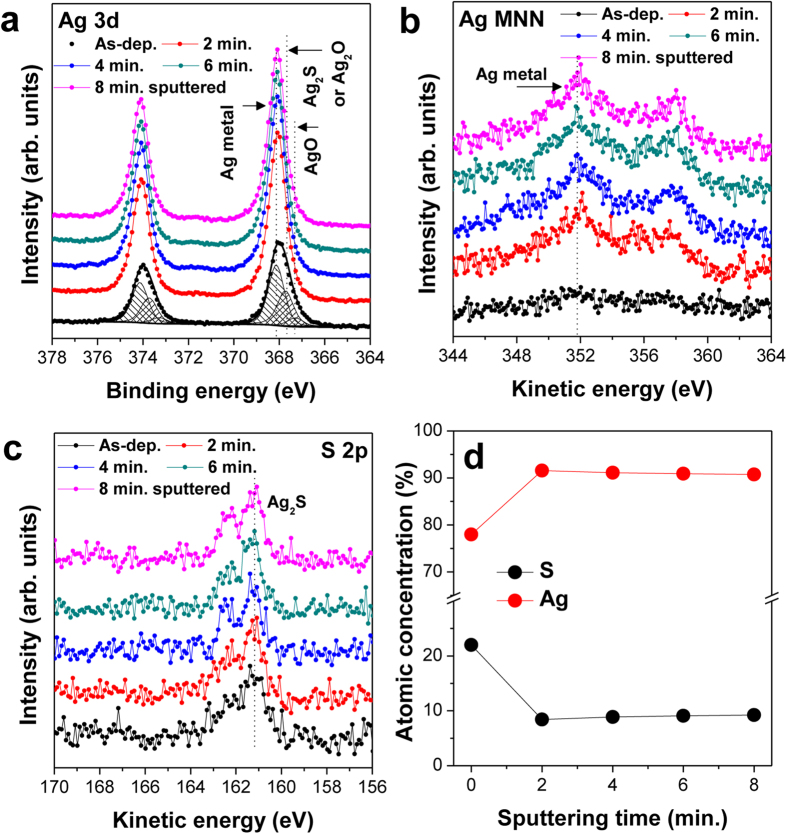
XPS depth profiles ((**a**) Ag 3d, (**b**) Ag MNN, and (**c**) S 2p) of denatured iGr/AgNW obtained by XPS measurement combined with Ar GCIB sputtering (5 kV, 2 × 2 mm^2^, and 2-min interval) depending on the Ar GCIB sputtering time. (**d**) The atomic concentration ratio of Ag and S atoms.

Although S was barely detected in the iGr-covered region, we cannot exclude the possibility that a sulfide layer may form on AgNWs underneath the iGr layer based on surface-sensitive techniques, such as XPS and AES without sputtering, alone. Furthermore, the typical sputtering technique using mono-atomic Ar^+^ ions is not an efficient method for iGr-covered AgNWs because the bare AgNWs were also intensely sputtered, thus preventing a meaningful comparison of the iGr-covered and bare AgNW regions. To selectively remove the iGr layer without severely damaging the AgNWs, we adopted the Ar GCIB sputtering technique, wherein low-energy sputtering is achieved with accelerated Ar clusters^42^,^43^. Using this gentle Ar GCIB sputtering technique, organic materials are sputtered with minimal damage and little sputtering of inorganic materials. To selectively sputter the iGr layer, we repeated the gentle Ar GCIB sputtering process using a low-beam-acceleration voltage and monitored the chemical information obtained by alternating XPS measurements. The XPS depth profiles and atomic concentrations according to the Ar GCIB sputtering time are given in Fig. 4a‒d. The optimized Ar GCIB sputtering conditions preferentially removed the iGr layer from the AgNWs without altering the AgNWs’ chemical/physical structures, and as a result, the AgNWs that were protected by iGr layers were exposed on the surface. Comparing the XPS spectra of iGr/AgNW samples before and after Ar GCIB sputtering allowed us to directly investigate the oxidation/sulfidation states of the AgNWs under the iGr layer. After iGr layer removal, the peak area of the Ag metal state (Ag 3d: 368.2 eV) increased, whereas those of the other states—Ag_2_S (S 2p: 161.3 eV) and AgO_x_ (Ag 3d: 367.7 and 367.1 eV)—remained constant. Additionally, as shown by the Ag MNN Auger and Ag 3d spectra, the increased Ag metal states do not decrease again, despite the continuous Ar GCIB sputtering process. These results indicate that during Ar GCIB sputtering, the AgNWs did not experience physical etching or chemical structure transitions. Therefore, the Ag:S component ratio increased and then remained constant as Ar GCIB sputtering progressed, as shown in Fig. 4d.

**Figure 5:**
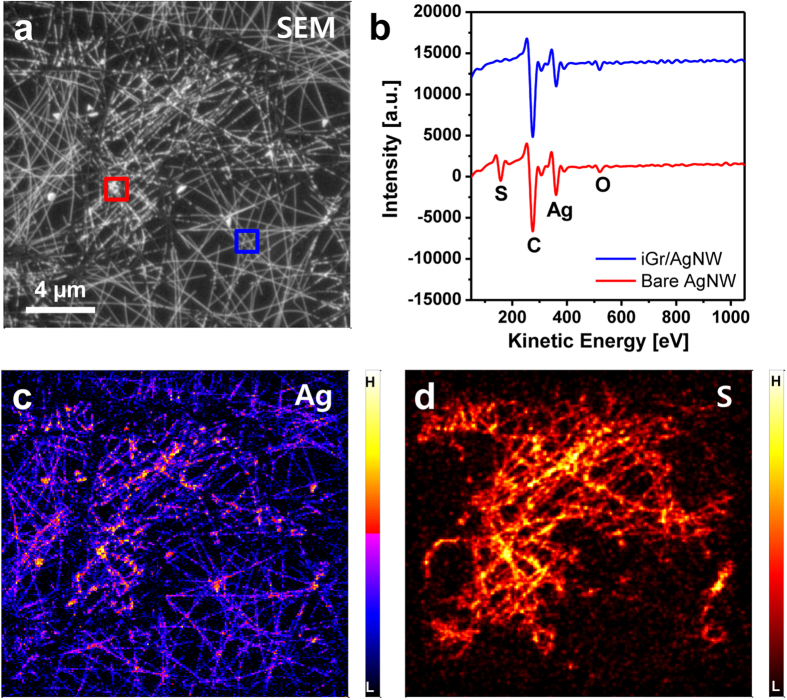
AES measurements of iGr/AgNW after 5 weeks with the selective removal of iGr layers by careful Ar GCIB sputtering: (**a**) SEM image, (**b**) AES spectra, and (**c**) Ag and (**d**) S mapping images.

Figure 5 presents the AES results of iGr/AgNW after the selective removal of the iGr layer via gentle Ar GCIB sputtering. No significant structural change was observed in the morphology shown in the SEM image (Fig. 5a), which supports the gentleness of the Ar GCIB sputtering technique, whereas a substantial difference is evident in the AES spectra (Fig. 5b) relative to those without sputtering (Fig. 3b). In Fig. 5b, the blue AES curve measured in the iGr-covered AgNW region shows the intense Ag peak which can be compared with the Ag peak of red AES curve measured in the bare AgNW region and it shows that the interference caused by iGr layer disappeared after the iGr layer was removed. This result was also confirmed by the Ag and S elemental mapping images (Fig. 5c,d), in which the Ag maps of the iGr/AgNW and bare AgNW regions are similar. In contrast, S was not imaged clearly in the iGr-covered region, even after the iGr layer was removed, but gave intense signals in the bare AgNW region. Based on these XPS and AES results obtained with Ar GCIB sputtering, we can conclude that the growth of sulfide in the iGr-covered AgNW region is negligible and that Gr is a highly efficient a barrier layer to protect AgNWs from corrosion via sulfidation when stored in the atmosphere.

**Figure 6:**
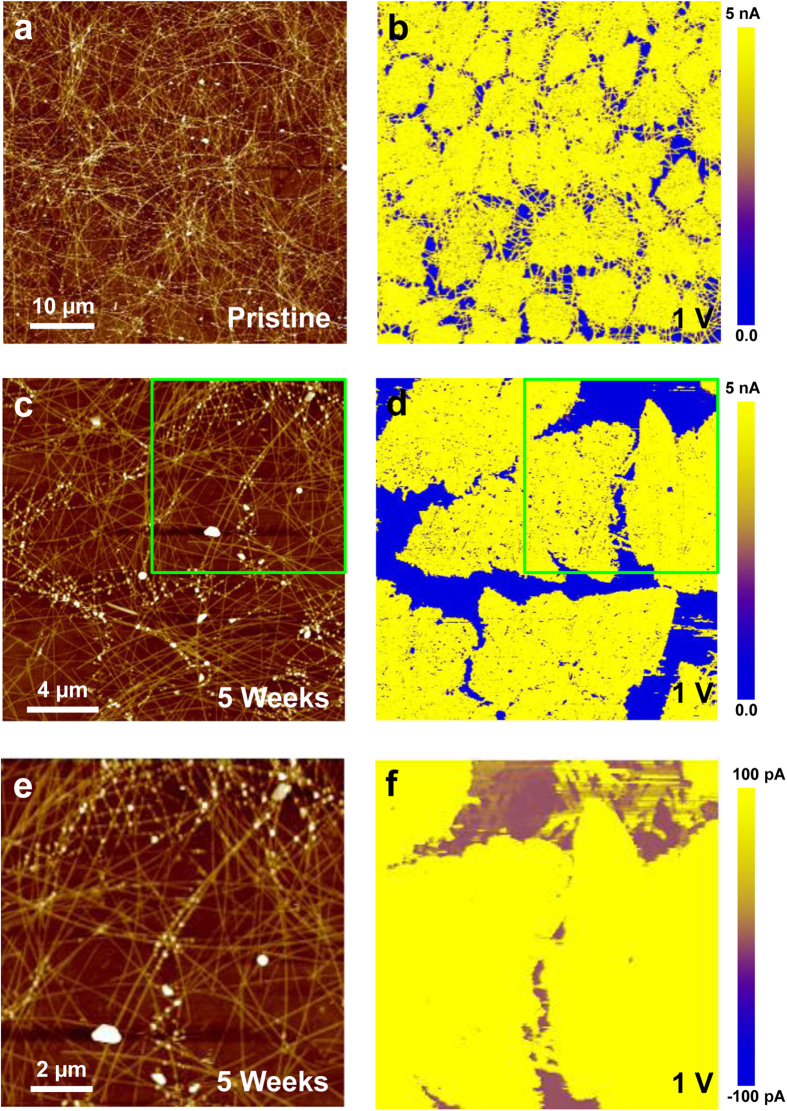
(**a**) Topographical and (**b**) current mapping images of pristine iGr/AgNW. (**c**,**e**) Topographical and (**d**,**f**) current mapping images of iGr/AgNW after 5 weeks. The sample bias voltage was 1 V in (**b**,**d**,**f**).

The corrosion of AgNWs is a critical issue affecting their application in TCEs because it induces electrical degradation and mechanical destruction, as shown in Supplementary Fig. 2. However, the electrical degradation of AgNWs can be hindered by covering them with Gr, as some research groups have already reported that large-scale electrical stability can be obtained by covering AgNWs with Gr layers based on long-term stability tests^26^,^27^. Through the electrical resistance and optical measurements for our bare AgNW, iGr/AgNW, and fGr/AgNW samples, we confirmed that the Gr-covered AgNW samples show remarkably improved electrical stability in air, while the decrease in transparency by Gr-covering is slight (~2.4% decrease for full Gr covering), as shown in Supplementary Fig. 4. According to the literature, the AgNW network samples protected by reduced graphene oxide (rGO) or graphene oxide (GO) have excellent electrical stability in the extreme environment of H_2_S gas exposure^44^‒^46^. In this study, we performed nanoscale electrical measurements via C-AFM to examine the nanoscale electrical stability of iGr/AgNW samples. In C-AFM, the nanoampere-scale current map between the tip and the sample is acquired simultaneously with a conventional AFM topographic image using a conductive AFM tip, as depicted in Supplementary Fig. 5a. Figure 6 shows the C-AFM results for the iGr/AgNW samples before and after undergoing considerable sulfidation. For the pristine iGr/AgNW sample shown in Fig. 6a,b, similar currents exceeding 5 nA flow through the iGr-covered and bare AgNW regions at a sample bias voltage of 1 V. Indeed, pristine AgNWs were more conductive than the iGr-covered AgNWs according to C-AFM because the iGr layer increases the resistance between the conductive AFM tip and the AgNWs in the vertical direction, even though the Gr layer is highly conductive and thin. The superior conductance of pristine AgNWs was demonstrated by the C-AFM measurements collected with a low bias voltage, as shown in Supplementary Fig. 6, in which greater current was measured on the pristine AgNWs than in the iGr-covered AgNW region. In Fig. 6c,d, the topographical and corresponding current images of the iGr/AgNW sample after long-term exposure to the atmosphere are shown. After undergoing considerable corrosion, including sulfidation, the conductance of the bare AgNWs was drastically degraded, as indicated by the barely detectable current on most of the bare AgNWs shown in Fig. 6d. In contrast, the iGr-coved AgNW region shows the conductance levels similar to those measured before corrosion. Indeed, most Gr islands were physically isolated in our samples, and the current measured in the iGr-covered AgNW region should also flow through the bare AgNWs. Therefore, the degraded AgNWs in Fig. 6c were not completely disconnected mechanically or electrically. In Fig. 6e,f, which present enlarged images of Fig. 6c,d using a different current scale (from ‒100 pA to 100 pA), a low current of ~100 pA was measured on the bare AgNWs, confirming that the bare AgNWs are not completely disconnected mechanically and electrically. The drastic degradation of these bare AgNWs arises from the growth of thin insulating layers, such as Ag sulfides and oxides, on their surfaces, although their inner regions still consist of Ag metal. The intermediate insulating layer between the AFM tip and the inner pure AgNW could create an additional resistance that far exceeds that of the extremely thin iGr layer. According to the literature, the nominal thickness of the Ag sulfide layer is a few nanometers after one month^12^,^17^; therefore, this layer could drastically reduce the electrical conductance between the AFM tip and the inner pure AgNW, as depicted in Supplementary Fig. 5b.

**Figure 7:**
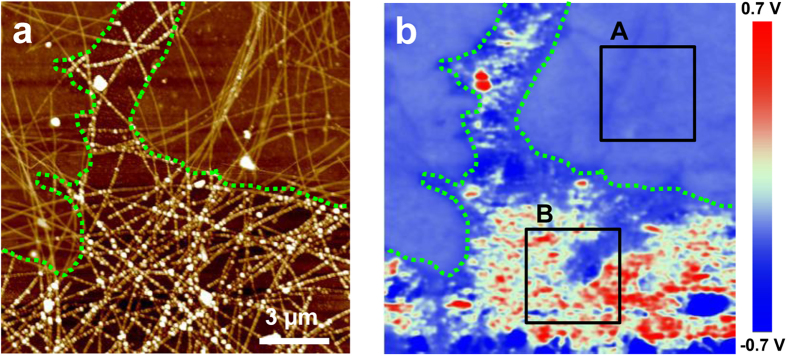
(**a**) Topographical image and (**b**) CPD map of iGr/AgNW after 5 weeks. The green dotted lines indicate the iGr layer on the AgNWs.

The work function or surface potential is a crucial property of candidate materials for TCEs because these materials frequently form heterostructures with the various organic or inorganic materials used in many applications, and the work function should be appropriately aligned with these other materials’ electronic structures, including their work functions, electronic bands, and molecular orbital levels. In addition to the nanoscale chemical and electrical stabilities of Gr-covered AgNWs, using Kelvin probe force microscopy (KPFM), we found that this Gr-AgNW hybrid system exhibits outstanding homogeneity in its surface potential. Among the various techniques that can be used to determine the surface potential or work function, including UV photoelectron spectroscopy (UPS), photoelectron spectroscopy in air (PEAS), and SEM with electron beam-induced current (EBIC), KPFM is a useful AFM-based technique that can determine the nanoscale distribution of surface potential with outstanding spatial resolution by measuring the contact potential difference (CPD) between the conductive AFM tip and the sample surface^47^. Figure 7 shows the topographical and corresponding CPD mapping images measured for the degraded iGr/AgNW sample. In Fig. 7b, the CPD is practically homogeneous in the iGr-covered AgNW region but fluctuates widely in the bare AgNWs region that experienced considerable corrosion. The homogeneity of the CPD can be compared quantitatively by acquiring its root mean square (RMS) values on the iGr-covered and bare AgNWs regions, respectively (the rectangular regions indicated as A and B in Fig. 7b). The RMS value of region A in Fig. 7b (~15 mV) is much smaller than that of region B (~350 mV). In addition, various methods of tuning Gr’s work function have already been thoroughly studied, and most methods are expected to be applicable to modulating the work function of the Gr-AgNW hybrid system.

**Figure 8:**
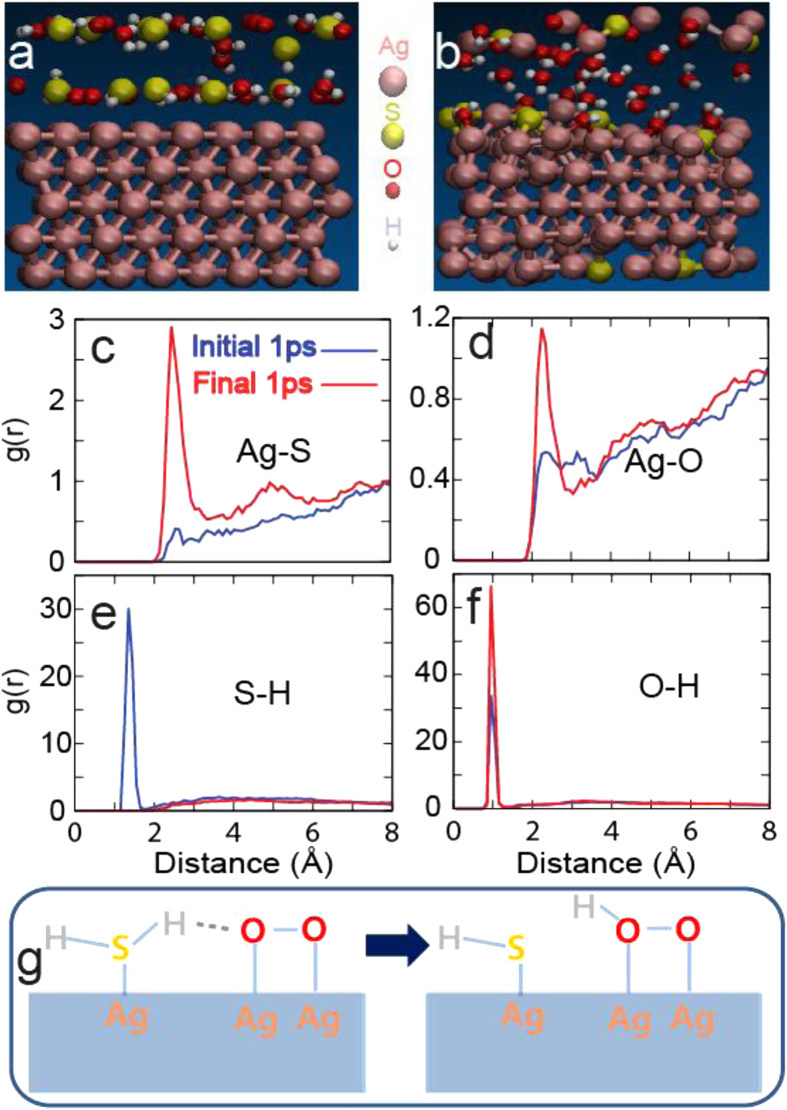
The geometries of the (**a**) initial and (**b**) final time steps, and the RDFs of the (**c**) Ag-S, (**d**) Ag-O, (**e**) S-H, and (**f**) O-H pairs. The initial (0–1 ps) and final (22–23 ps) 1-ps datasets are used to demonstrate how the surface reactions reorganized the chemical bonds. (**g**) The schematic shows the mechanistic pathway for the dehydrogenation of H_2_S on the Ag surface.

To gain more comprehensive atom-scale insights, we also performed *ab initio* molecular dynamics (AIMD) simulations based on density functional theory (DFT) using the Vienna *Ab* initio Simulation Package (VASP)^48^. Because of the limitations on the size that AIMD can handle, we focused on the Ag(111) surface rather than the NW geometry. As a sulfidation source, 12 H_2_S gas molecules were considered, with the same numbers of O_2_ and H_2_O, which are abundant under ambient conditions, considered as potential oxidants. After geometry relaxation of the gas molecules on the Ag(111) surface (Fig. 8a), we performed MD at 1000 K to observe reactions within AIMD’s limited timescale. After a simulation time of 23 ps, bonding rearrangement occurred, as shown in Fig. 8b. At this point, the sulfidation of the Ag surface is evident, and the radial distribution functions (RDFs) presented in Fig. 8c‒f allow clear visualization of the behaviors of the chemical reactions over time. The RDFs for the initial (blue) and final (red) 1-ps simulations show how atoms changed their coordinations. The strong enhancement of the first peak indicates a significant increase in the bonding peak of the Ag-S pair. However, only mild increase occurred in the first peak in the RDF of the Ag-O pair. The RDFs of the S-H and O-H pairs clarify why the tendency toward sulfidation is stronger than that for oxidation. The strong first nearest-neighbor peak in Fig. 8e for the initial 1-ps simulation corresponds to the bonds in H_2_S molecules. However, after 23 ps, all of the H atoms that were originally attached to S are absent, instead bonding with O and enhancing the first peak in the RDFs of the O-H pair, as shown in Fig. 8f. The schematic diagram in Fig. 8g presents the mechanistic pathway of the reaction that led to the sulfidation of the Ag surface. H atoms originally bonded to S transfer to O_2_, generating H_2_O and surface OH. The transfer of H is analogous to the transfer of a proton through hydrogen bonds in water. In the gas phase, hydrogen bonds cannot form between H_2_S and O_2_ because the O atom is charge neutral. However, when O_2_ adsorbs on the Ag surface, it acquires a negative charge similar to that of O atoms in H_2_O by withdrawing an electron from the surface. Thus, a hydrogen bond forms between the O_2_ and the Ag surface, which facilitates the dehydrogenation of H_2_S. We observed that in the absence of H, S atoms react more vigorously with the surface Ag. Thus, some surface Ag atoms are pulled off of the surface, and S penetrates into the Ag substrate.

## Conclusion

In conclusion, using a novel analytical approach, we confirmed that Gr can act as a barrier layer to protect AgNWs against atmospheric corrosion, especially sulfidation, in the Gr-AgNW hybrid system. Furthermore, the Gr layer efficiently protects the underlying AgNWs from sulfidation, and the growth of Ag sulfide on the protected AgNWs was found to be negligible after selectively removing the Gr layer via Ar GCIB sputtering. In addition, nanoscale current mapping demonstrated the outstanding electrical stability of Gr-covered AgNWs and the drastic degradation of the electrical conductance of bare AgNWs after atmospheric corrosion. Finally, AIMD explained the strong tendency toward sulfidation by H_2_S, even in the presence of possible oxidant gases, such as O_2_ and H_2_O, and revealed this process’s mechanistic pathway.

## Methods

### Experimental Details

Fabrication process for iGr/AgNW hybrid films: Commercially available chemical vapor deposition (CVD) monolayer iGr (Gr coverage on Cu foil of 40~50% according to optical microscopy) on Cu foil (15 × 15 cm) was purchased from GRAPHENE SQUARE Co. (South Korea). The poly(methyl methacrylate) (PMMA) solution (20 mg/mL) was spin-coated onto iGr/Cu foils at 3,000 rpm for 30 s and dried in air overnight. Fe chloride (97%, Sigma Aldrich) was used to etch Cu, and the PMMA/Gr film was floated on the surface of the deionized (DI) aqueous solution. The PMMA/Gr film was placed in distilled water several times to remove the etchant residue and finally floated on a large bath for wet transfer to the AgNW networks.

Commercially available aqueous AgNW dispersions (Aiden Co. Ltd., South Korea) with NW diameters of ~20 nm and lengths of 15–25 μm, were redispersed in water:ethanol cosolvent (70:30 volume ratio) at a concentration of 0.1 mg mL^−1^ with hydroxypropyl methyl cellulose (2,600‒5,600 cP, Sigma Aldrich) as a binder (10wt./wt.% relative to the amount of AgNWs). AgNW networks with the targeted sheet resistance of ~30 Ω/□ were wire-bar coated on transparent, flexible polyethylene terephthalate (PET) film (KIMOTO 100 CPB, Japan) with a thickness of 100 μm. Then, the AgNW films were annealed for 5 min in a convection oven at 100 °C.

To create the iGr/AgNW hybrid film, the AgNW-coated films were placed into PMMA/iGr-floated DI water and then slowly lifted from one side so that the PMMA-coated Gr sheet covered the top of the AgNW electrode. Subsequently, the PMMA/iGr/AgNW/PET(or PI) was gently dried with a blow dryer from the back. Then, the PMMA coating was dissolved in acetone, and the acetone was refreshed three times to completely remove the PMMA. Finally, the iGr/AgNW films were annealed for 5 min in a convection oven at 100 °C to establish stable contact between iGr and the AgNWs. To observe the degradation of iGr/AgNW in the atmosphere, the samples were stored in the laboratory for several weeks (temperature and RH of approximately 25 °C and 30% RH, respectively).

Scanning probe microscopy (SPM) measurements: All AFM measurements were collected utilizing ambient SPM (Dimension Icon, Bruker) in C-AFM and KPFM modes. PtIr or conductive diamond-coated AFM tips purchased from NanoWorld AG for the C-AFM and KPFM measurements. We performed conventional AFM imaging in tapping mode with the simultaneous acquisition of phase images to clearly distinguish the iGr layer on the iGr/AgNW sample, and the C-AFM experiments were conducted with a nominal force of 100 nN and a slow line scan rate of 0.2 Hz for the stable acquisition of current mapping images, as shown in Supplementary Fig. 5a. Typical AFM and C-AFM results of pristine AgNWs with the sample bias voltage changed from −1 V to 1 V are shown in Supplementary Fig. 1a,c,d.

XPS and AES measurements: The XPS and AES experiments were conducted using an ultrahigh vacuum (UHV) XPS instrument (PHI 5000 VersaProbe, ULVAC-PHI) with Ar GCIB sputtering equipment and a UHV scanning Auger nanoprobe (PHI 710, ULVAC-PHI), respectively. The gentle Ar GCIB sputtering was performed with a beam voltage of 5 kV and a raster size of 10 × 10 mm^2^, and the chemical structure of each sample was investigated during the sputtering process via XPS. The Ar GCIB-sputtered sample was transferred rapidly into the AES chamber using a vacuum transfer vessel able to maintain a low vacuum level and minimize sample contamination during transfer. The AES measurements were conducted with a beam voltage and current of 3 kV and 10 nA, respectively.

### DFT Calculation Details

Projector augmented wave (PAW) potentials were used with a planewave basis cutoff of 400 eV. We considered the semicore d-levels of Ag as valence. The Perdew-Burke-Ernzerhof (PBE) exchange-correlation functional was used with Greme’s scheme for van der Waals interactions^49^. To mimic the surface, five layers of Ag were used, and the atoms in the central layer were set to not move during MD. The supercell size was 11.556 × 20.016 × 20 Å. To speed up the MD simulation, we used gamma point sampling. The tritium mass was used for H to allow setting a 1-fs time step.

## Additional Information

**How to cite this article**: Kim, S. H. *et al.* Nanoscale Chemical and Electrical Stabilities of Graphene-covered Silver Nanowire Networks for Transparent Conducting Electrodes. *Sci. Rep.* **6**, 33074; doi: 10.1038/srep33074 (2016).

## Supplementary Material

Supplementary Information
