# Supplementary material for: Nanoscale Chemical and Electrical Stabilities of Graphene-covered Silver Nanowire Networks for Transparent Conducting Electrodes
Source: Sci Rep. 2016 Sep 13;6:33074. doi: 10.1038/srep33074 (PMC5020617; doi:10.1038/srep33074)
Supplement: Supplementary Information [file srep33074-s1.pdf]

Supplementary Information for

Nanoscale Chemical and Electrical Stabilities of Graphene-covered Silver Nanowire Networks for Transparent Conducting Electrodes

Seong Heon Kim[1], Woon Ih Choi[1], Kwang Hee Kim[1], Dae Jin Yang[1], Sung Heo[1]

& Dong-Jin Yun[1]*

[1]Samsung Advanced Institute of Technology, Gyeonggi-do 443-803, Republic of Korea

* Corresponding author: Dongjin.yun@samsung.com

Keywords: silver nanowire, sulfidation, graphene, gas barrier, gas cluster ion beam (GCIB) sputtering

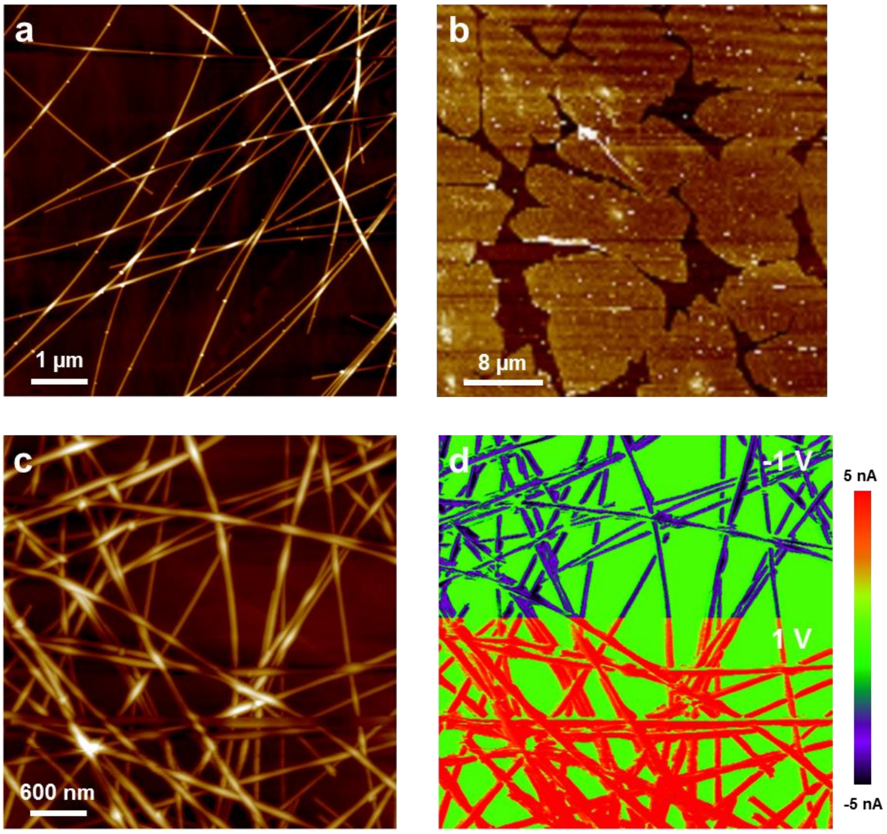

Supplementary Figure 1: (a), (b) Topographic images of (a) AgNWs and (b) iGrs. (c) Topography and (d) current mapping images of bare AgNWs with changing sample bias voltage from -1V to 1V.

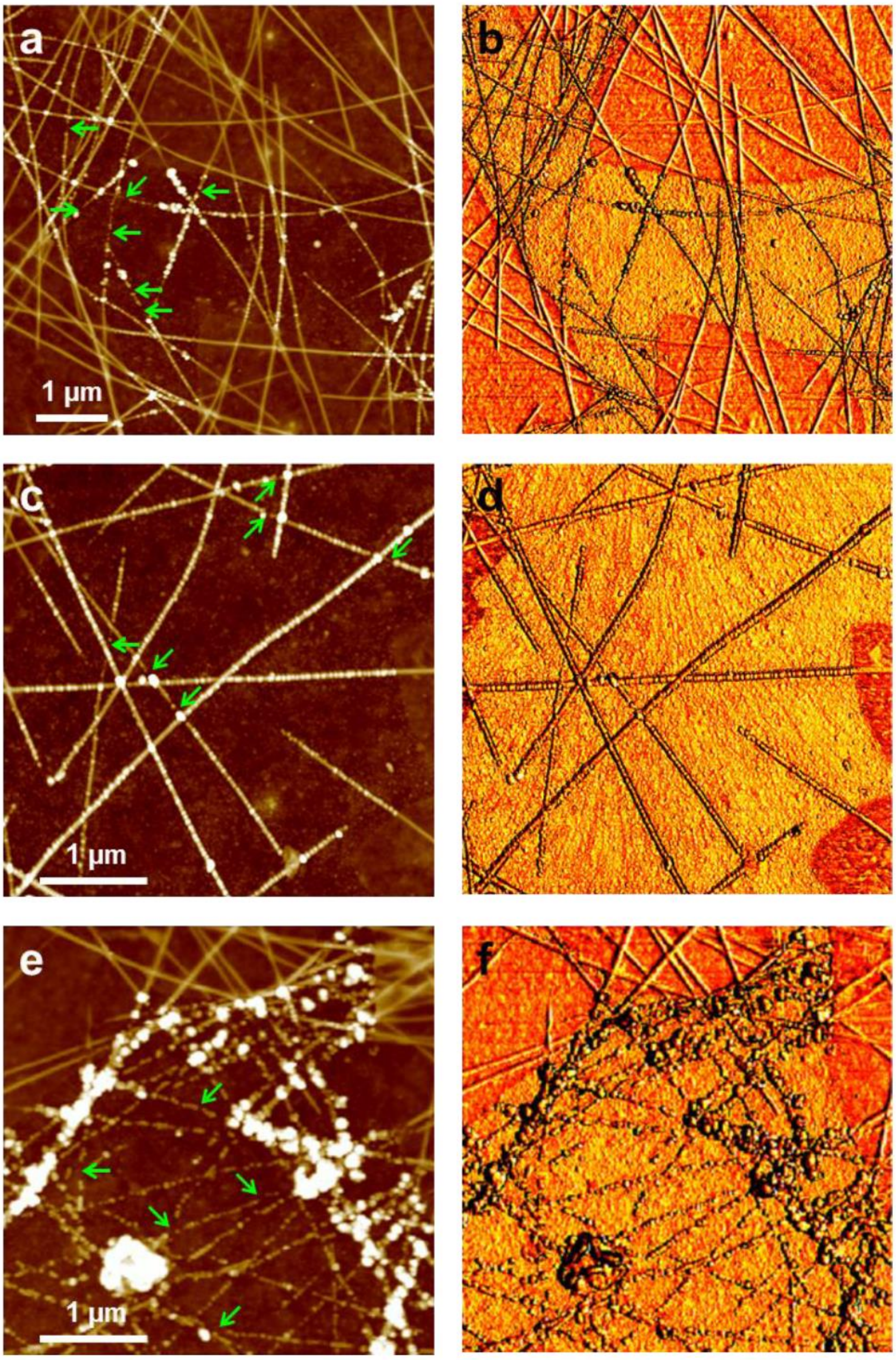

Supplementary Figure 2: (a), (c), (e) Topographic and (b), (d), (f) phase images of iGr/AgNW after 8 weeks. The green arrows in (a), (c), (e) indicate the selected points where AgNWs are ruined and disconnected.

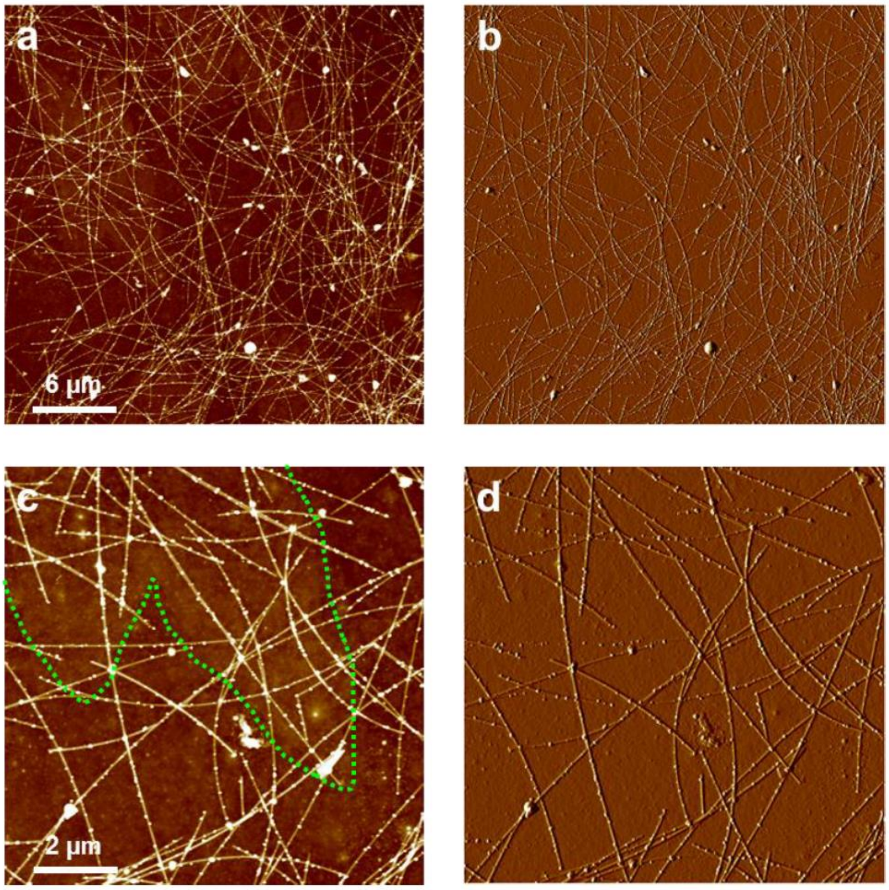

Supplementary Figure 3: (a), (c) Topographic and (b), (d) differentiation images of AgNW/iGr after 3 weeks. The green dotted line in (c) indicates the iGr layer under AgNWs.

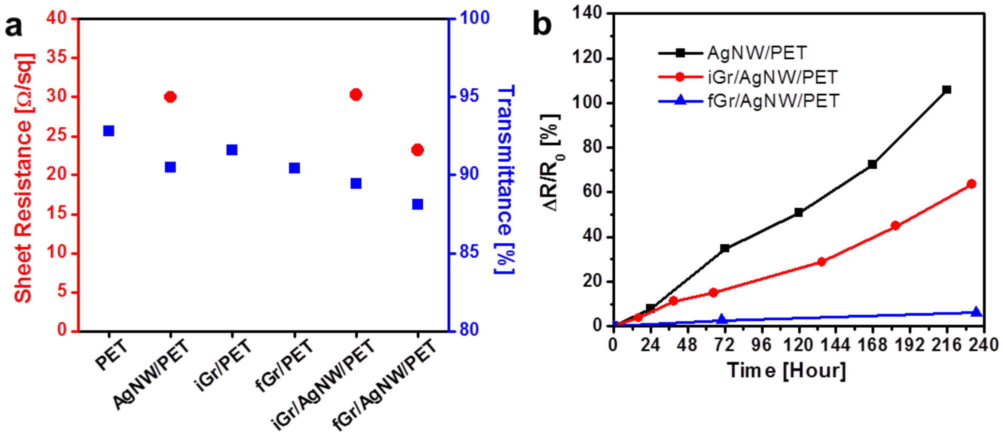

Supplementary Figure 4: (a) Initial sheet resistance (red circles) and optical transmittance (blue squares) measurements for homogenous or hybrid films of AgNW, iGr, and fGr. (b) Sheet resistance changes ($\Delta R/R_0$) for AgNW/PET, iGr/AgNW/PET, and fGr/AgNW/PET samples.

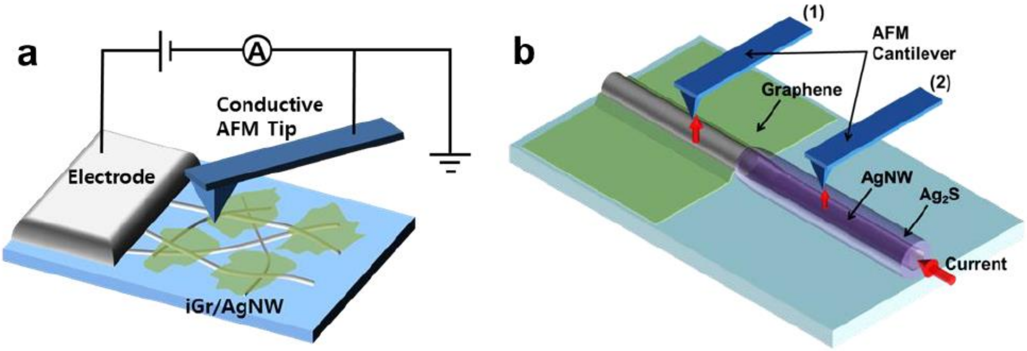

Supplementary Figure 5: (a) Schematic of C-AFM measurement setup for iGr/AgNW. (b) Schematic of current flow with different AFM tip positions. The AFM tip contact position (1) and (2) indicates the iGr-covered and the sulfidized part of AgNW, respectively.

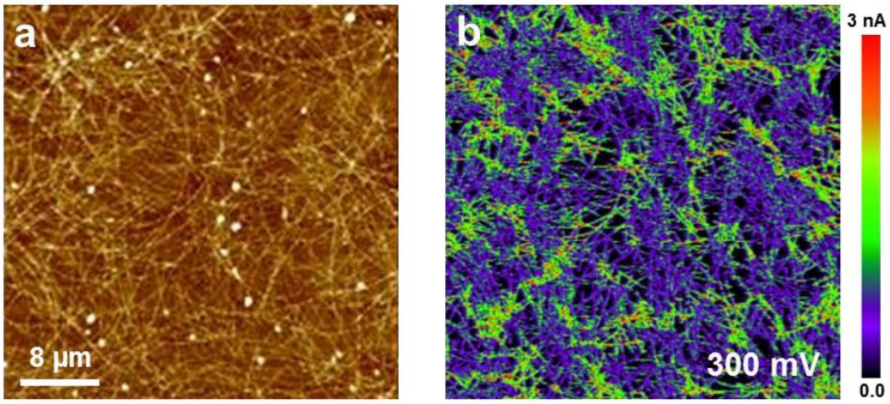

Supplementary Figure 6: (a) Topography and (b) current mapping images of pristine iGr/AgNW. The bias voltage is 300 mV in (b).
